# Appendico-Ileal Knotting: A Rare Cause of Strangulated Small Bowel Obstruction

**DOI:** 10.4314/ejhs.v34i2.7

**Published:** 2024-03

**Authors:** Najeem Adedamola Idowu, Waheed Olalekan Ismaeel, Akeem Aderogba Adeleke, Joshua Adejare Faleye, Suliyat Adebisi Adeleye-Idowu, Kehinde Aderonke Ademoye

**Affiliations:** 1 Urology division, Department of Surgery, Ladoke Akintola University of Technology Teaching Hospital and LAUTECH Ogbomoso; 2 Department of Family Medicine, Geriatric centre, Obafemi Awolowo University Teaching Hospital, Complex Ile-Ife; 3 Department of Surgery, General Surgery Unit, Obafemi Awolowo University Teaching Hospital Complex Ile-Ife; 4 Ministry of Health, Oyo State, Nigeria; 5 Department of family medicine, Jericho Specialist Hospital Ibadan; 6 Department of Physiology, Obafemi Awolowo University Ile-Ife

**Keywords:** appendix, strangulated small bowel, ileal knot

## Abstract

**Background:**

Bowel obstruction is a common surgical emergency worldwide. It may result into high morbidity or mortality whenever intervention is delayed. It affects all age groups. The most commonly seen etiologies of bowel obstruction are post-operative adhesions, neoplasm and hernia in that order. Intestinal knot syndrome is an extremely rare cause of intestinal obstruction, and when it occurs, it poses diagnostic challenges. We report a case of appendico-ileal knotting causing strangulated small bowel obstruction due to its rarity and diagnostic difficulty. Our objective is to discuss the clinical presentation and management of this rare cause of surgical emergency.

**Case:**

A-72- year old man was seen at the emergency unit of our center with 4 days history of gradual onset of colicky abdominal pain with nausea and vomiting. He had a two-day history of constipation and a one-day history of fever. He was acutely illlooking and his vital signs were abnormal. Urgent abdominopelvic ultrasound and plain abdominal x-ray was performed and were suggestive of intestinal obstruction. He had emergency laparotomy, and intra-operatively appendico-ileal knotting was seen with gangrenous appendix and terminal ileum. This necessitated limited right hemicolectomy and ileo-colonic anastomosis. The patient was managed post-operatively and discharged on post-operative day 10.

**Conclusion:**

Appendico-ileal knotting is a cause of small bowel obstruction although it is very rare. The diagnosis is commonly confirmed intra-operatively. There are reports of simple small bowel obstruction secondary to appendico-ileal knotting, but this case confirmed that it could also lead to strangulated intestinal obstruction.

## Introduction

Intestinal obstruction is a common surgical emergency in the world and appendico-ileal knotting is one the rare causes of this surgical condition (1). Appendico-ileal knotting may be defined as an impediment to the flow of intestinal contents such as fluid, entericus and gases. Intestinal obstruction may be simple when the blood supply to the obstructed segment is intact or strangulated when the blood supply to the obstructed part is compromised Patients with bowel obstruction, including appendico-ileal knotting usually present in emergency unit. They will require immediate intravenous fluid resuscitation, parenteral antibiotics and analgesia administration. Urgent abdominopelvic ultrasound and plain abdominal radiograph, erect and supine are required to establish the diagnosis of bowel obstruction including appendico ileal knotting . Some patients with appendico ileal knotting causing bowell obstruction may require advance cross sectional imaging especially if malignancy is being suspected or in a situation of ambiguity In some cases, diagnosis of appendico ileal knotting causing bowel obstruction may only be confirmed intra-operatively. Intestinal knot syndrome is an extremely rare cause of intestinal obstruction and when it occurs, it poses diagnostic challenges. Some of the intestinal knot syndrome causing intestinal obstruction that have been reported in the medical literature include ileoileal knotting, ileosigmoid knotting, ileal cecal knotting and appendico-ileal knotting among others. Appendico-ileal knotting, otherwise known as appendicular tie syndrome or appendicular tourniquet, is the rarest of them all.

We report a case of appendico-ileal knotting causing strangulated small bowel obstruction due to its rarity and diagnostic difficulty. Our objective is to discuss the clinical presentation and management of this rare cause of surgical emergency.

## Case

**Figure 1:**
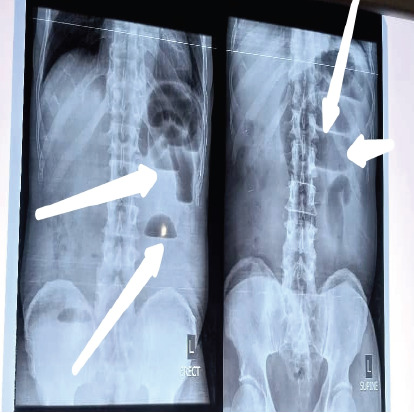
Plain abdominopelvic radiograph showing multiple air-fluid level as indicated by the white arrow

**Figure 2:**
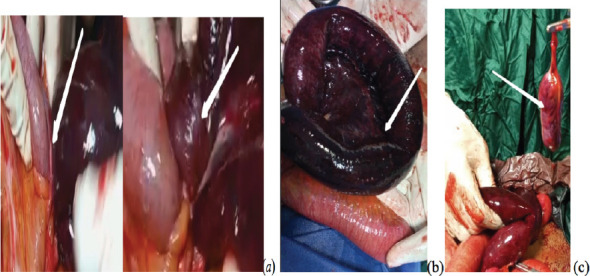
(a) showing intra-operative findings with an arrow indicating knot formed by the appendix wrapping around a loop of small bowel, an arrow indicating the gangrenous part of the appendix before surgical removal; (b) an arrow indicating gangrenous small bowel; (c) an arrow indicating gangrenous appendix following surgical removal

A 72-year-old man was seen at the emergency unit of our center with 4 days history of gradual onset of colicky, persistent abdominal pain with nausea and vomiting. He had a two-day history of constipation and a one-day history of fever. There was an associated history of abdominal distension. He had no history of weight loss or hematochezia. There was no previous history of surgical operation or antecedent trauma to the abdomen. He had neither history of hypertension nor diabetics or other co-morbidities. He was acutely illlooking, and his vital signs were abnormal. He had intravenous fluid resuscitation. Parenteral antibiotics and analgesia were also commenced immediately. Urgent abdominopelvic ultrasound and plain abdominal x-ray (erect and supine as shown Figure 1) were done which were suggestive of intestinal obstruction. A preoperative diagnosis of a suspected acute malignant bowel obstruction was made. Complete blood count revealed pack cell volume of 39%, white blood cell count of 14,000 and 76% neutrophilia. Electrolyte, urea and creatinine were; sodium of 140mmol/l, potassium of 3.9mmol/l, chloride of 101mmol/l, bicarbonate of 22mmol/l, urea of 4mmol/l and creatinne of 85mmol/l. All these fell within the normal range. He had emergency laparotomy and intra-operatively appendico-ileal knotting was seen with gangrenous appendix and terminal ileum within 40cm from ileo-cecal junction (Figure 2). The patient had limited right hemicolectomy and ileo-colonic anastomosis. He was discharged on post-operative day 10.

## Discussion

Intestinal knot syndrome is a surgical condition in which two segments of the bowel become twisted together. It may involve only the small bowel such as Ileo-ileal knot, or it may be between small and large bowe such as Ileo-sigmoid knotting, Ileo-cecal knotting and appedico-Ilea knotting. Appendico ilea knotting also, referred to as appendicular tie syndrome, is an extremely rare surgical conditions

It has been classified as true knot in which a knot is formed following a loop created by another part of the bowel and as pseudo-knot in which there is presence of loop but no knot is formed. To the best of the authors' knowledge, this is the second case of appendicular tie syndrome to be reported in Nigeria and one of the few cases known in the literature. Ajiboye et al reported the first case in 2017 (3). There have been more reports of ileoseigmoid and ileocecal knotting compared to appendicular tie syndrome. KoheiKammari et al reported the 13^th^ case of Ileo –Ilea knotting in the literature Issam knotiing in a series Yazough et al also reported three cases of ileosigmoid knotting (4). This could not be said to be of appendico-ileal knotting. Patients with intestinal knot syndrome are commonly seen in emergency with acute abdomen whether it is simple or strangulated bowel obstruction. This index case was not an exemption as the patient was observed with strawasngulated bowel obstruction. Appendicoileal knotting can affect all age groups although it is commonly seen in the 3^rd^ decade of life Chandan et al had also reported a case of appendico-ileal knotting in a 26-year-old man. The occurrence of this pathology in the elderly as observed in this case has further established that it can affect all age group.

Appendico-ileal knotting is seen commonly in males as widely reported in the literature The reason for slight male preponderance is largely unknown However, it may not be unconnected to men socioeconomic activities that gives little or no time for regular intake of high fiber diet. The incidence of intestinal knot syndrome is said to be lower in the west. This is probably linked to high intake of high fiber diet.

The presumptive diagnosis of intestinal obstruction may be made following history of abdominal pain and most commonly nausea and vomiting with or without abdominal distension and constipation. There will be multiple air fluid level on plain abdominopelvic radiograph erect and supine views. However, the underlined etiology is often confirmed intra-operatively because there may not be enough time for advanced cross-sectional imaging to establish the diagnosis pre-operatively due to urgent need for surgical exploration to reduce morbidity and mortality. This case was not an exemption as diagnosis of intestinal obstruction was suspected from history and radiological evidences of obstruction. Although malignant bowel obstruction was our pre-operative diagnosis due to old age of the patient when malignancy is said to be much more common. More so, malignancy is the third most common cause of bowel obstruction after postoperative adhesions and hernia. The patient did not, however, present with other constitutional symptoms of malingnancy. This diagnostic dilemma may be resolved by advanced cross-sectional imaging such as abdominal computed tomography and magnetic resonance imaging, especially in patient not in acute presentation. This was similarly reported by Issamu et al in their work on the discovery of gangrenous appendix and part of terminal ileum intra operatively. The appendix completely wrapped around the loop of the terminal ileum. There will be initial lymphatic obstruction followed by venous obstruction and subsequently ischemic necrosis as noted intra-operatively. This will lead to intestinal perforation and generalized peritonitis due to release of intestinal contents into the peritoneum. However, perforation was not observed in this case, probably due to quick intervention. Some reported in their series of normal appendix as well as viable intestine while others had similar observation as in this case The surgical treatment of appendico-ileal knotting depends on intra-operative findings. In this case, limited right hemicolectomy plus ileocolonic anastomosis was done because of gangrenous appendix and terminal 40cm part of ileum up to the cecum. The second option we had was to do ileal resection and ileo-ileal anastomosis because the gangrenous ileum only spared the cecum and by principle cecum cannot be anastomosed with ileum in a septic situation.

This informed our choice of limited right hemicolectomy. It has been reported in some cases that derotation and appendectomy may suffice in cases of viable bowel (5).

Appendico-ileal knotting is a cause of small bowel obstruction although. It is very rare. The diagnosis is commonly confirmed intra-operatively. There are reports of simple small bowel obstruction secondary to appendico-ileal knotting, but this report has shown/confirmed that it could as well lead to strangulated bowel obstruction.
